# Autonomous CE Mass‐Spectra Examination for the Ocean Worlds Life Surveyor

**DOI:** 10.1029/2022EA002247

**Published:** 2022-10-18

**Authors:** Steffen Mauceri, Jake Lee, Mark Wronkiewicz, Lukas Mandrake, Gary Doran, Jack Lightholder, Zuzana Cieslarova, Miranda Kok, Maria F. Mora, Aaron Noell

**Affiliations:** ^1^ Jet Propulsion Laboratory California Institute of Technology Pasadena CA USA

**Keywords:** science autonomy, onboard summarization, science utility estimation, Europa, Enceladus

## Abstract

Ocean worlds such as Europa and Enceladus are high priority targets in the search for past or extant life beyond Earth. Evidence of life may be preserved in samples of surface ice by processes such as deposition from active plumes, hydrofracturing, or thermal convection. Terrestrial life produces unique distributions of organic molecules that translate into recognizable biosignatures. Identification and quantification of these organic compounds can be achieved by separation science such as capillary electrophoresis coupled to mass spectrometry (CE‐MS). However, the data generated by such an instrument can be multiple orders of magnitude larger than what can be transmitted back to Earth during an ocean world’s mission. This requires onboard science data analysis capabilities that summarize and prioritize CE‐MS observations with limited computational resources. In response, the autonomous capillary electrophoresis mass‐spectra examination (ACME) onboard science autonomy system was created for application to the ocean world’s life surveyor (OWLS) instrument suite. ACME is able to compress raw mass spectra by two to three orders of magnitude while preserving most of its scientifically relevant information content. This summarization is achieved by the extraction of raw data surrounding autonomously identified ion peaks and the detection and parameterization of unique background regions. Prioritization of the summarized observations is then enabled by providing estimates of scientific utility, including presence of key target compounds, and the uniqueness of an observation relative to previous observations.

## Key Points

- Onboard science autonomy makes high data rate instruments viable for interplanetary missions with limited bandwidth
- Developed onboard summarization and prioritization of data from CE‐MS raw files to compress raw mass spectra by orders of magnitude
- Prioritization of samples by science utility estimate and diversity allows to maximize science yield

## 1 Motivation

The search for extraterrestrial life is one of the great motivators for exploring worlds beyond Earth. Ocean worlds, such as Europa and Enceladus, offer protected, potentially habitable environments that may be sampled from the surface through inclusions in thermally convected ice, pressure driven fluid through fractures, or deposition by active plumes, for example (Carr et al., 1998; Postberg et al., 2009; Quick et al., 2017). To prepare for such deep space missions to these icy worlds, NASA's Jet Propulsion Laboratory (JPL) is developing the ocean worlds life surveyor (OWLS), an in‐situ instrument suite capable of detecting multiple, independent biosignatures indicative of life. At the molecular scale, terrestrial life may be detected by the presence of key organic compounds such as metabolites and amino acids. However, as extant life may have resulted from a separate genesis, an in‐situ instrument must be sensitive to as broad a spectrum of life‐like molecules as possible. This challenging analytic goal must be achieved on an ice sample, using only the limited computation available to space missions, and in an autonomous fashion (Howell et al., 2020; Tan‐Wang & Sell, 2019; Theiling et al., 2022; Willis et al., 2020).

To enable the detection of molecular‐scale evidence of life, OWLS includes a capillary electrophoresis (CE) electrospray ionization mass spectrometry (MS) instrument (Mora et al., 2022). The combination of CE and MS technologies provides a two‐dimensional fingerprint that can be used to uniquely identify and quantify a wide range of molecular species. Sample molecules are first separated by CE, producing migration times that vary according to the ratio of molecular size to charge. Then, the MS breaks molecules into unique fragmentation patterns separated by their mass‐to‐charge (*m/z*) ratio. The resulting observations appear as a two‐dimensional grid of ion counts, superimposed on a complex noise background. The OWLS suite includes a chemical sensing unit that characterizes the samples, for example, pH, conductivity, before analysis. Recent work has shown that the CE‐MS instrument can be used to detect a wide range of biomolecules in liquid samples (Mora et al., 2022), even in the presence of large concentrations of dissolved salts such as may be present on both Europa and Enceladus (Hibbitts et al., 2019; McCord et al., 1999; Postberg et al., 2011; Trumbo et al., 2019).

The extreme distance between ocean worlds and Earth severely limits the amount of data that can be returned due to the energy required for transmission, the limited power available onboard, and the availability of the deep space network. For example, the entire downlink budget for a reference mission to Europa (Tan‐Wang & Sell, 2019) is roughly the same size as a raw CE‐MS samples (approximately 190 MB). Given that multiple, independent samples from multiple instruments will be needed to characterize a landing site (Lindensmith et al., 2022), a space‐born CE‐MS instrument must be able to reduce its observations by at least two orders of magnitude, to be feasible for mission inclusion. This compression cannot be performed agnostic to the science use‐case if valid science conclusions are to be preserved; the detailed, high‐frequency structures within the ion peaks must be captured, preventing application of simple Fourier or Wavelet filters or lossy image formats such as JPEG. Rather, onboard summarization capabilities must be developed to support the precise scientific analyses that will be performed once data is returned to Earth. Even with effective onboard summarization, the desire to fully characterize the surface will drive science teams to consider as many samples as possible. To support representative sampling and robust characterization, a further onboard capability to prioritize among these summarized samples is highly desirable. This ensures that high‐value observations are returned first as measured by quality (strong signal to noise), evidence for compounds of known interest, and uniqueness with respect to previous observations. The autonomous capillary electrophoresis mass‐spectra examination (ACME) science autonomy software (Machine Learning and Instrument Autonomy Group, Jet Propulsion Laboratory, 2022) provides both summarization and prioritization capabilities to meet these needs.

## 2 Hardware and Data

### 2.1 Instrument Description

The CE‐MS experiments used for ACME's development and evaluation were optimized for the goal of broadly separating a wide variety of biological compounds relevant to the search for life in the presence of confounding environmental salts (Mora et al., 2022). Briefly, CE‐MS was carried out on a CESI 8000 instrument (SCIEX, Brea, CA) coupled to a 3D quadrupole ion trap mass spectrometer (LCQ Fleet MS), equipped with a nanospray MS source (Thermo Electro North America LLC, Madison, WI). Separations were performed using bare fused silica capillaries (91 cm long with a 30 μm I.D.) with a porous tip (OptiMS cartridge, SCIEX), and acetic acid as background electrolyte. As described in Mora et al. (2022) two concentrations of acetic acid were studied: 2 and 5 M. The 5 M solution was selected as optimal because it is more tolerant to high concentrations of salts. Samples were hydrodynamically injected using a pressure of 2 psi for 20 or 60 s, corresponding to an injection volume of approximately 7 and 21 nL, respectively. Larger injection volumes were typically used at low organic and low salt concentration samples to achieve greater signals when high salt content was not present. Analytes were separated by applying a voltage of 20 kV and 2 psi of pressure at the inlet of the capillaries, and the capillary temperature was set at 25°C. Data were acquired using positive ionization mode in the mass range of 70–400 *m/z*. Refer to Mora et al. (2022) for a complete description.

### 2.2 Input Data Description

**Figure 1:**
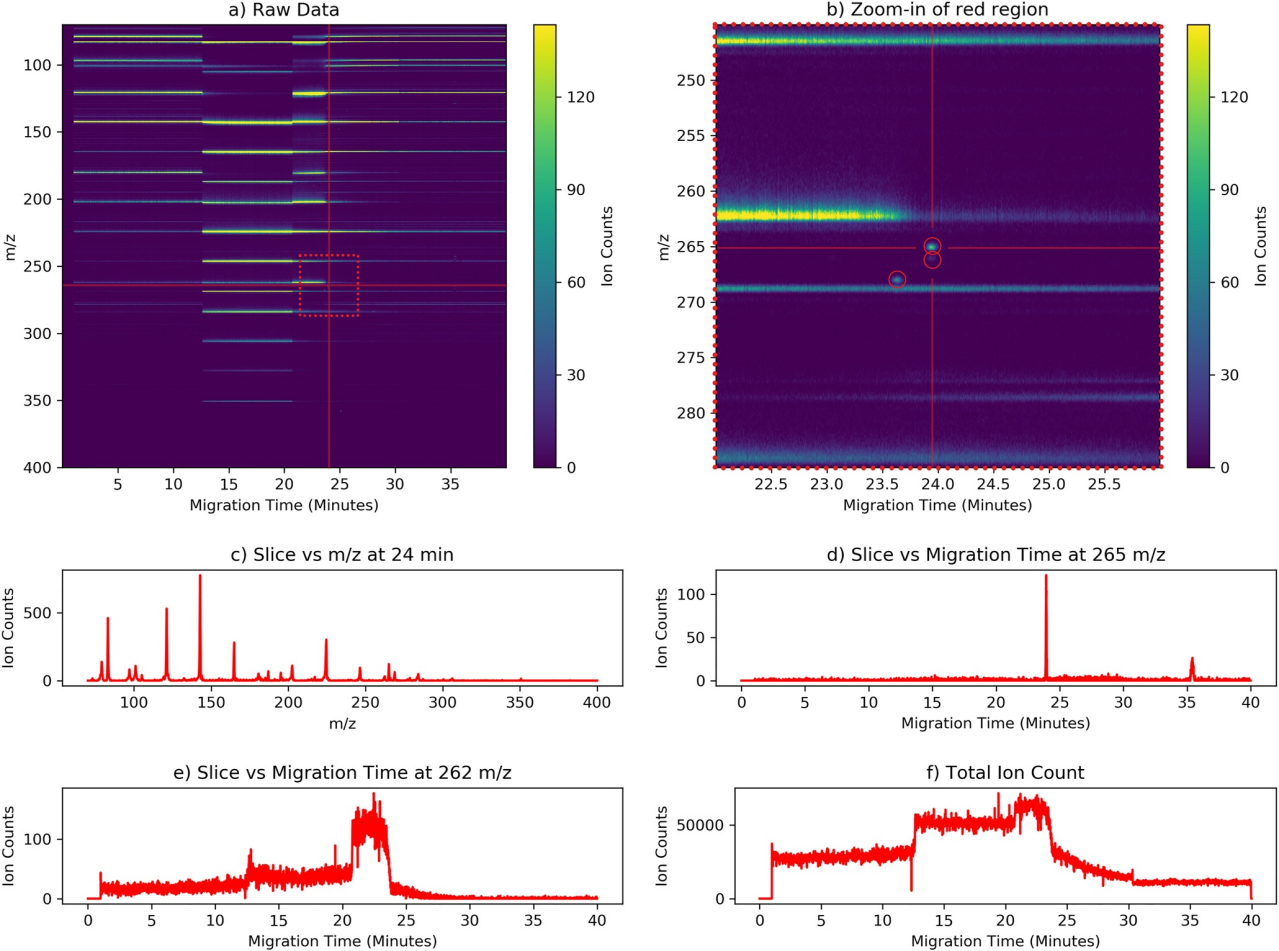
Example of raw data from a sample with 10 uM of Mix25 (25 organic compounds relevant to astrobiology studies) in a 3 M NaCl solution. (a) Entire 2D ion count grid resolved by migration time (*x*‐axis) and mass‐to‐charge ratio (*y*‐axis). Horizontal structures are due to the presence of salt in the sample and are part of the background. Individual peaks are too small to be visible. Note, legend bar is cropped to increase contrast. (b) Zoom‐in of the red box showing individual peaks (circled in red) and cliff‐like, horizontal “salt fronts.” (c) Mass spectra corresponding to a migration time of 24 min ±10 s. (d) Electropherogram plotting ion count versus time for the mass bin *m/z* = 265, showing a peak at 24 min. (e) Electropherogram for *m/z* = 262 showing a “salt front.” (f) Electropherogram plotting total ion count versus time.

The OWLS CE‐MS instrument outputs 2D grids of raw ion counts resolved by their *m/z* and migration time as shown in Figure 1. The *m/z* resolution of 0.08 amu is constant over the full range. The temporal resolution varies between 0.3 and 0.5 s, depending on the instrument operation mode. Each analyzed sample produces approximately 100 MB of raw data with 5,000 *m/z* bins and 4,000 time bins. By summing over a specified *m/z* dimension, an electropherogram may be generated consisting of the total ion counts in the specified mass range versus time (A special case is the “total ion count” electropherogram that reduces the data back to a 1D time separation by summing over the entire mass range). The heights of peaks in these electropherograms correspond to the concentration of the parent compound. Similarly, slicing the data at a single migration time produces an associated mass spectra (ion counts vs. *m/z*). The migration time is related to the mobility of a compound under an electric field. The total scientific information content of a CE‐MS observation corresponds to identifying and characterizing all ion count peaks in the 2D data in the presence of a potentially complex, noisy background. This noise can originate from random instrument fluctuations, regions with elevated and variable ion counts, and high concentrations of salts.

#### 2.2.1 Laboratory Samples

ACME's development incorporated nearly 2,000 samples produced in the laboratory environment, concurrent with the development of the CE‐MS instrument itself (Mauceri et al., 2022). A standard set of 25 organic compounds relevant to astrobiology (L‐leucine, L‐alanine, *β*‐alanine, L‐histidine, glycine, L‐valine, L‐serine, L‐aspartic acid, L‐glutamic acid, *γ*‐aminobutyric acid [GABA], 2‐aminoisobutyric acid [AIB], Gly‐Gly, Gly‐Gly‐Gly, Leu‐Leu‐Leu, Phe‐Val, cytosine, adenine, guanine, uracil, cytidine, adenosine, guanosine, thymidine, uridine, and isovaline) (Creamer et al., 2017; Mora et al., 2022; Neveu et al., 2018), referred to as Mix25, was analyzed at various concentrations and in the presence of a range of salt concentrations to serve as baselines with known peak locations. These analyses were performed in close coordination with instrument scientists, facilitating a tight iterative loop that enabled the parallel development of the CE‐MS instrument and the associated ACME science autonomy system. An example separation of Mix25 is shown in Figure 1.

#### 2.2.2 Data Set Annotation

##### 2.2.2.1 Development Set

This data set was used for ACME's algorithm selection, development, and to inform and evaluate the instrument hardware development. Ion count peaks that represent the target science observables were annotated by hand in the raw data. The annotated data set spans eight independent samples varying in their concentration of Mix25 (100 nM–90 μM) and the amount (0–3 M) and type of salt (NaCl and MgSO_4_), as described in (Mora et al., 2022). To capture the annotations, 471 electropherograms per sample were produced by binning the ion counts every 0.7 *m/z*. The MATLAB Computer Vision Toolbox's Image Labeler (MathWorks, 2022) was used to manually annotate the time range for every peak with a z‐score (peak height over baseline divided by the local noise environment) greater than 5. Annotations were performed and reviewed by both instrument scientists and the autonomy team's data scientists. Instrument scientists identified valid peaks by considering a combination of factors such as peak shape, migration time, expected peak width with respect to its migration time, and the signal‐to‐noise ratio. The total annotated data set comprises eight independent samples with 907 labeled peaks.

##### 2.2.2.2 Training Set

After finalizing the ACME algorithm using the development set, a second labeled data set was prepared to optimize ACME's filtering parameters as detailed in Section 3.2 as well as discourage over‐fitting to the limited samples in the development set. The training set extends the development set by seven samples that varied concentrations of Mix25, NaCl, and MgSO_4_ salts, and acetic acid. Hand annotation was performed by an instrument scientist using Xcalibur (Thermo Scientific, 2022), a proprietary mass spectrometry analysis software by Thermo Fisher, and verified by the autonomy team's data scientists. The training set consists of a total of 536 labeled peaks.

##### 2.2.2.3 Testing Set

To estimate ACME's generalized performance, an additional six, independent, lab‐prepared samples were produced that spanned Mix25 and NaCl concentrations as well as two injection volumes. As described in (Mora et al., 2022), no significant differences for known masses were observed between the two salts so NaCl was used as a model salt for experiments with low concentrations of organics. Annotations were produced as described in the training set. The testing data set contains a total of 292 labeled peaks.

Characteristics for each of these datasets are summarized in Table 1.

**Table 1:** Development, Training, and Testing Data Set Characteristics

Data set	Mix25 (uM)	Salt type	Salt conc. (M)	HAc conc. (M)	Injection vol. (nL)	# Labeled peaks
Development	0.1	‐	‐	5	21	5
	2	‐	‐	5	21	35
	5	NaCl	3	5	7	17
	10	NaCl	3	5	7	16
	50	MgSO_4_	0.15	2	7	235
	50	NaCl	0.15	2	7	155
	50	NaCl	0.6	2	7	232
	50	NaCl	1	2	7	213
Training	50	‐	‐	2	21	113
	50	‐	‐	5	7	81
	50	MgSO_4_	1.5	5	7	96
	50	NaCl	3	5	7	72
	50	NaCl	3	2	7	114
	2	NaCl	3	5	7	13
	2	‐	‐	5	21	47
Testing	0.1	‐	‐	5	21	15
	1	‐	‐	5	21	58
	10	‐	‐	5	21	88
	1	NaCl	3	5	7	10
	10	NaCl	3	5	7	19
	90	NaCl	3	5	7	102

#### 2.2.3 Simulated Data

**Figure 2:**
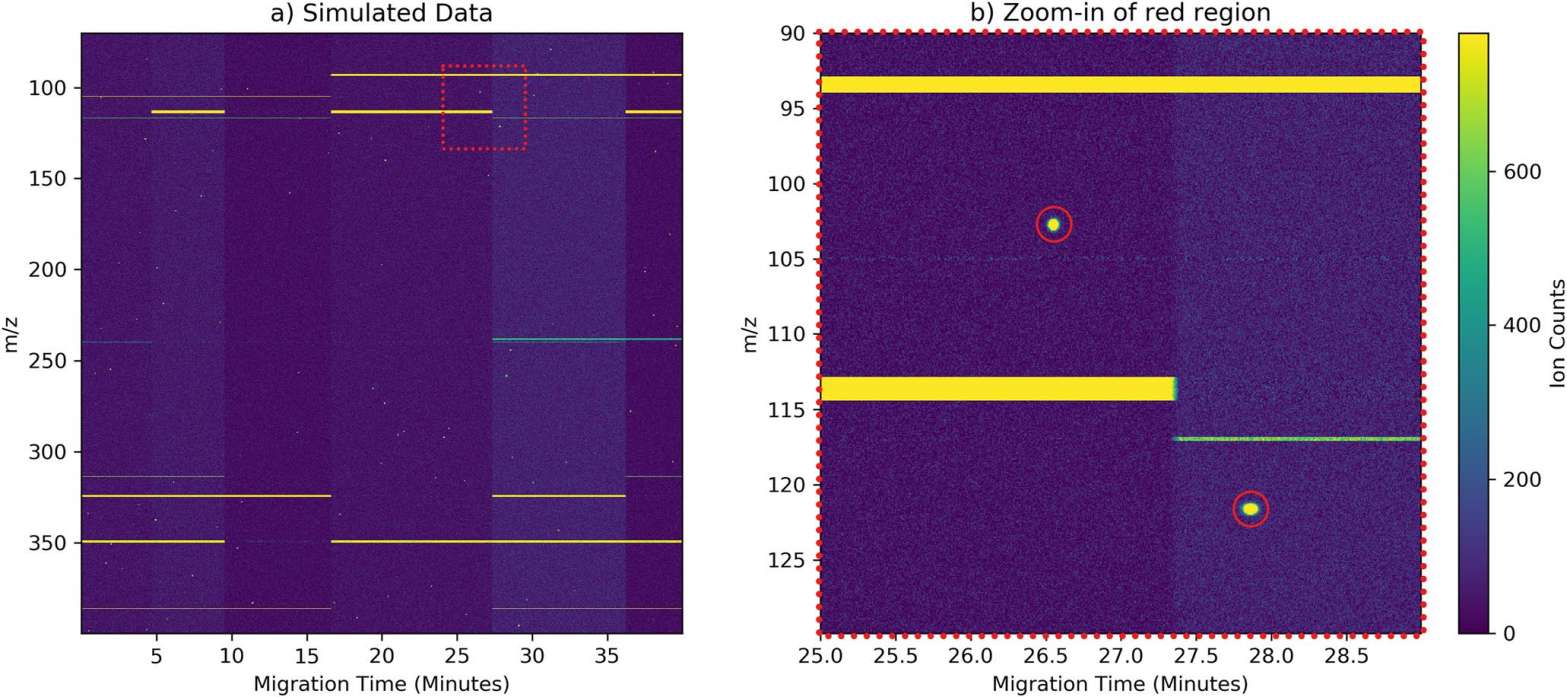
Example of simulated ion counts versus migration time and *m/z* for a sample from the “Silver” simulated data set: (a) Regions of differing noise characteristics and salt fronts are visible, (b) Zoom‐in to highlight individual peaks (circled in red).

To provide a highly controlled environment for performance evaluation, sensitivity analysis, and explore challenging separation scenarios, a CE‐MS data simulation capability was created. The simulator includes the ability to construct differing regions of noise characteristics and embed 2D Gaussian‐shaped target peaks with custom heights and widths. The simulator was used to create two evaluation data sets. The first, named “Golden,” was intended to test ACME's performance under ideal data conditions. It contained 20 simulated samples of 100 peaks per sample with *z*‐scores greater than 10 and an absence of complex background features such as salt fronts and regionally varying noise. The second data set, named “Silver,” more closely resembles lab‐provided instrument data and contains varying levels of background noise, horizontal salt front features, and peaks with *z*‐scores greater than 5. An example sample from the Silver data set is shown in Figure 2. While these data sets are simpler than the actual instrument data, they represent an unambiguous truth source that does not depend on human subjectivity for annotation. Here, we use both labeled laboratory data as well as simulated data to evaluate ACME's performance.

## 3 Methods

**Figure 3:**
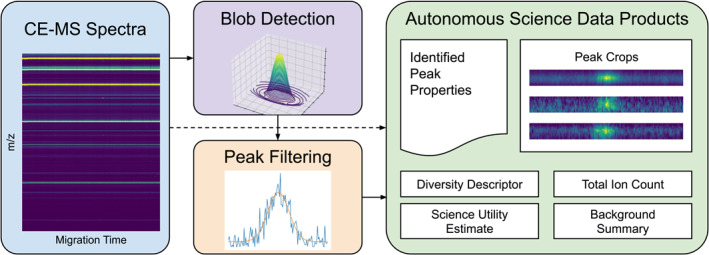
System level diagram of ACME. Left: raw data is collected using the CE‐MS. Center: ion count peaks are identified with blob detection from raw CE‐MS data. Identified peak candidates are filtered based on calculated peak properties. Right: finally, various autonomous science data products are generated and ranked for transmission to the ground.

The ACME processing pipeline for a new CE‐MS observation is structured into three major steps. First, ACME identifies and characterizes peaks in the raw CE‐MS ion count data (Section 3.2). Next, ACME extracts and compresses the scientifically relevant information for both peak and background regions that are used to generate several summary autonomous science data products (ASDPs) for potential downlink to mission control (Section 3.3). Finally, ACME enables later downlink prioritization among several observations either by the presence of high‐quality peaks of interest (the science utility estimate [SUE]) or by the presence of unusual or unique data features (the diversity descriptor [DD]) (Section 3.4). This system level description of ACME is captured in Figure 3.

### 3.1 ACME Reconfiguration and Operations

For application to a specific mission, the initial parameters that control ACME's behavior will be determined as described in the following sections using annotated data prepared similarly to Section 2.2. This initial set of parameters, or configuration, will enable pre‐flight validation of key performance requirements. However, once launched into space, a variety of influences may require alteration of ACME's initial configuration.

Several factors within the spacecraft and instrument may effectively lower the signal‐to‐noise ratio of the science observables. Flight instrument performance characteristics may change due to mechanical shocks and vibrations inherent during launch, degradation of sensor elements over the mission lifetime, accumulation of debris and contamination, and failures of the supporting systems necessary for proper instrument functioning such as temperature regulation and clean power supply. These factors may introduce complex background features and false peak‐like structures as well as suppress valid target peaks. The deployment environment may also differ from expectation in challenging ways. Noise due to radiation or the significant presence of uninteresting compounds with similar and confounding separation patterns could reduce data quality.

Even in the absence of these challenges, a science team's focus is likely to change. Once ubiquitous compounds of interest have been well characterized in a deployed environment, it is reasonable to shift a mission's attention to other more subtle or rare signals that may have differing peak characteristics than the primary mission targets used to tune ACME.

Whether in reaction to internal or external challenges, or to accommodate an evolving science focus, ACME has been constructed to be readily reconfigurable with the uplink of a small set of intuitive parameters (described throughout Sections 3.2 and 3.3). In a mission scenario, ground teams comprised of scientists and autonomous instrument operators will need to determine and validate new configurations prior to uplink. The team would capture new training data sets that incorporate any emergent data challenges and annotate them to emphasize the desired change in focus of ACME's summarization and prioritization.

### 3.2 Peak Detection Algorithm

The most scientifically valuable information in CE‐MS data corresponds to the location of all ion count peaks, expressed as a pair of mass‐to‐charge ratio and migration time. Reliably detecting and characterizing subtle peaks in the presence of a complex and varying background forms the primary function of ACME. If the peaks can be well identified and characterized, the resulting list fully describes a 100 MB data set of raw CE‐MS data using only approximately 10 kB and is agnostic to the specific compounds sampled. That is to say, by choosing to initially characterize peaks rather than search for specific compound signatures, the ability to discover unanticipated molecular species is preserved.

The onboard science autonomy use‐case presents unique requirements that sharply differ from laboratory data analysis in terms of computational constraints, robustness to unanticipated inputs, and interpretability (Slingerland et al., 2021). For example, while abundant open source software packages exist to find peaks in mass spectrometer data such as OpenMS (Sturm et al., 2008), XCMS (Smith et al., 2006), CWT (Du et al., 2006), MZmine2 (Pluskal et al., 2010), or more recently deep learning (Liu et al., 2019; Zhao et al., 2021), most of these algorithms are too computational expensive for space‐borne applications. ACME is designed to operate onboard a spacecraft with limited computational power (e.g., a 200 MHz radiation‐hardened processor (BAE Systems, 2017)) in real‐time, analyzing and summarizing a fresh sample without regular user guidance for parameter adjustment. Furthermore, the data products produced by ACME must support and enable rigorous scientific interrogation in lieu of access to the full raw data record. As the presence of biosignatures on an ocean world would be an extraordinary claim, ACME's results must also be highly interpretable and support confirmatory analysis on the ground. Further, summarization and prioritization decisions must be backed up by sufficient context to provide justification as well as raise alarms should a reconfiguration be required. This includes not only peak information but also descriptions of the complex background that may include unanticipated but mission critical structure. Toward these goals, ACME was developed utilizing a readily interpretable expert system for target peak detection that borrows concepts from many leading peak detection algorithms, strongly optimizes for computational efficiency, and captures snapshots of raw data around each identified peak to enable detailed ground analysis, re‐processing, and overlapping lines of evidence.

Rather than accuracy, the space use‐case bandwidth constraint places individual requirements on both false positive and false negative peak detections. False positive peak detections result in the high resolution capture of uninteresting portions of the CE‐MS ion count grid. These cause no harm so long as all target peaks are also captured. However, should false positive peaks become so numerous as to crowd out true positives for limited downlink bandwidth, valid science targets may not be included. False negatives, or missed peaks, whether by a failure in the peak detection step or by crowding out by false positives, may directly result in failure to capture key science observables. Using values from the Europa reference mission, produced ACME requirements for a false negative rate of less than 5% for peaks with *z*‐score greater than 5 and less than 50 false positive peaks per observation.

**Figure 4:**
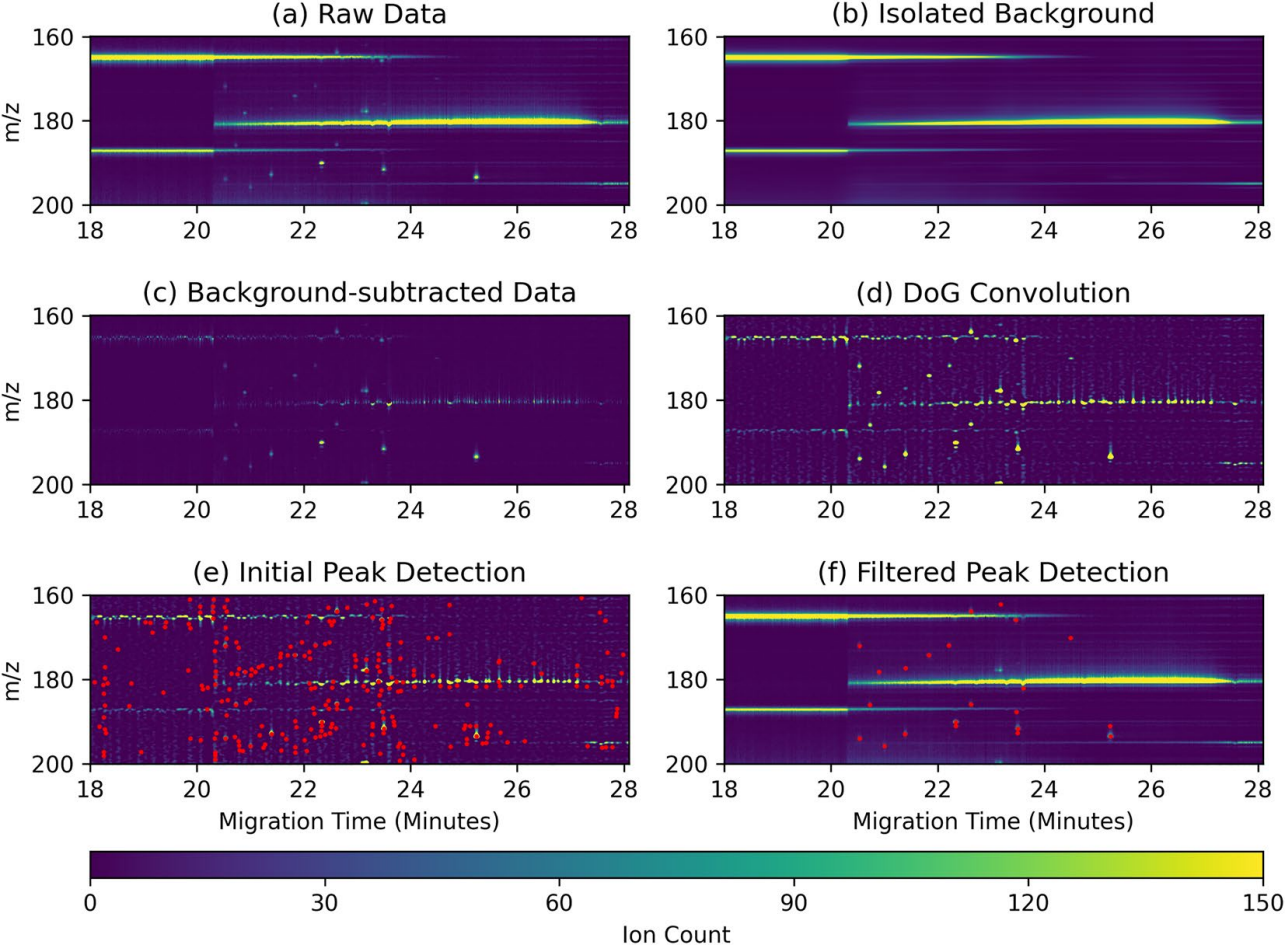
Steps of the peak identification algorithm on a cropped sample with 90 μM of Mix25 and 3 M NaCl (introduced in Section 2.2.1): (a) Raw data, (b) Background estimate from median filter, (c) Raw data with background subtracted, (d) Data is convolved with a Difference of Gaussians filter, (e) Peaks identified with non‐maximum suppression (shown as red dots), (f) Identified target peaks that were down selected from all found peaks by their properties (shown as red dots).

The peak detection algorithm steps are illustrated in Figure 4 and documented below. Generally, they proceed by first producing a low‐quality list of candidate peaks using computationally efficient methods, followed by a more sophisticated analysis of each candidate to separate valid from spurious peaks.

#### 3.2.1 Background Isolation

Raw data from the CE‐MS instrument typically includes distinct and slowly varying regions of elevated, noisy background. These regions are visible as horizontal streaks in Figure 4a, and are often influenced by salt concentrations. In the same figure, target peaks can be seen as bright dots; the location of these peaks must be identified by isolating them from the noisy background. To estimate this background, a median filter is applied along the migration time axis to smooth over the noise. Large filter window sizes produce higher quality background estimates, but induce errors near abrupt changes in background behavior. On the other hand, small windows produce noisy background estimates and may confuse valid, wide peaks as background noise. An optimal median filter window size of 36 s was determined by maximizing performance on the training data set. Figure 4b shows an example of an isolated background estimate. By subtracting this estimate from the raw data and setting negative values to zero, the peaks are isolated from their local noisy background.

#### 3.2.2 Initial Peak Candidate Detection

In preparation for the blob detection process, the isolated peaks are convolved with a Difference of Gaussians (DoG) spatial filter that is produced by subtracting a two‐dimensional Gaussian from a smaller‐width Gaussian. Intuitively, this selects for peak‐like structures that have a Gaussian shape and suppresses structures that deviate from such a shape, as this was found to well represent the valid CE‐MS targets of interest. The DoG filter is defined by setting the SD of the two Gaussian filters in both the mass and time dimensions. An example result of enhanced peaks is shown in Figure 4d.

Non‐maximum suppression blob detection is then applied to find the local maxima of the now isolated potential peaks. The resulting list of peak maxima contains valid peaks embedded among far more numerous false‐positive, spurious noise peaks. An example of peak identification results is shown as red dots in Figure 4e.

#### 3.2.3 Peak Characterization

**Figure 5:**
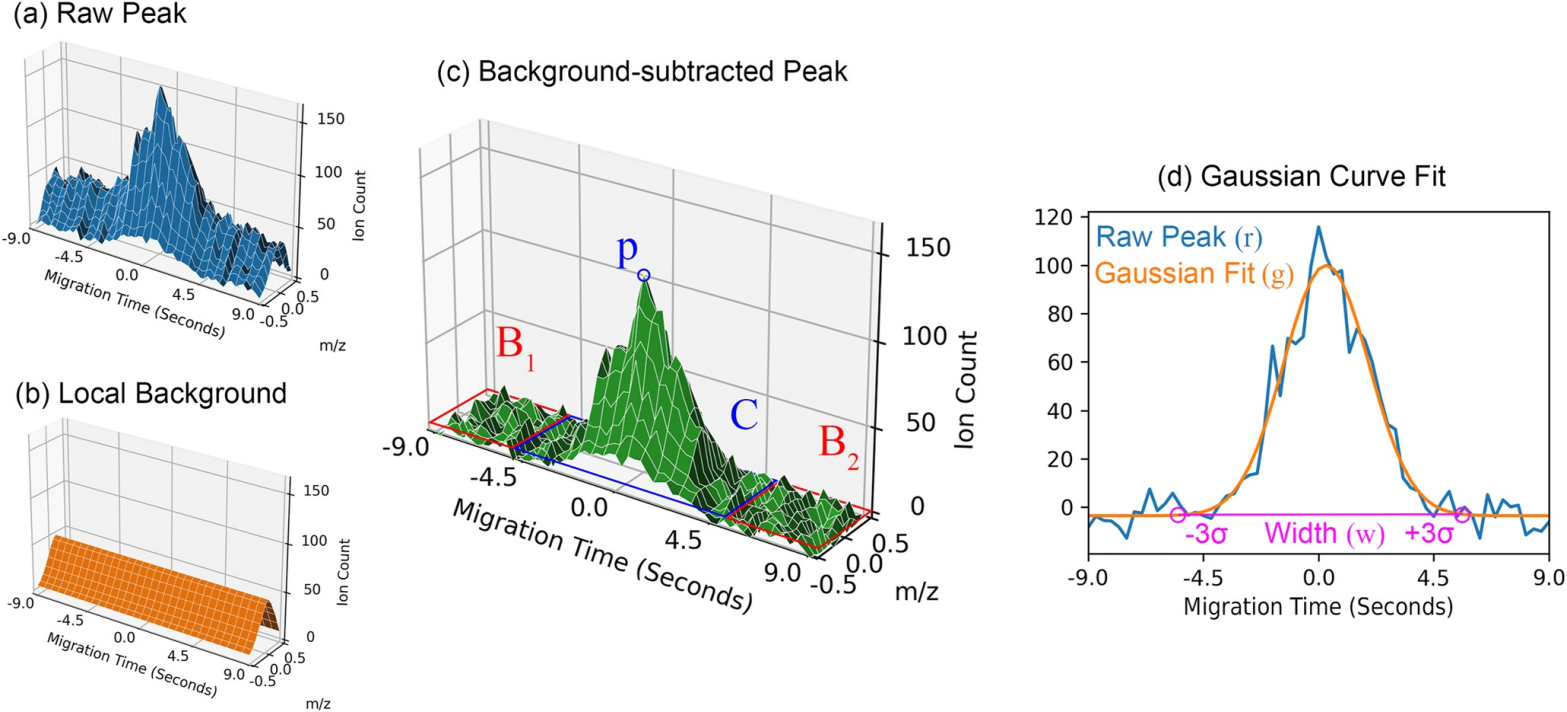
Core elements of calculated peak properties. The cropped peak from raw CE‐MS data is shown in (a), and the estimated local background is shown in (b). Subtracting (b) from (a) results in (c), which is the isolated signal. Labeled variables include the surrounding background region, *B*_1_ and *B*_2_, central peak region, *C*, peak height, *p*. (d) The central *m/z* slice of (c), with a Gaussian fit, *g*, of *r*, and peak width, *w*.

Valid target peaks may be separated from spurious peaks based on detailed examination of their properties in the original raw ion count data. To extract these properties, the local region surrounding each peak candidate (Figure 5a) is first examined to estimate a local background (Figure 5b) that is removed by subtraction (Figure 5c). The peak's width *w* and Gaussianity is estimated using a Gaussian fit to the central *m/z* slice of the windowed region (Figure 5d). With this information, the peak's height, volume, width, and Gaussian loss can be calculated to quantify the shape of the peak. Further, defining the temporal region before the peak (*B*_1_), the peak itself (*C*), and after the peak (*B*_2_), background features such as the noise level, SD, and the difference/ratio of the noise before and after the peak can be calculated. These features are formally described in Table 2. These peak properties were captured from the manual data investigations used by instrument scientists during instrument development and characterization.

**Table 2:** Extracted Peak Properties

Peak property	Equation	Description
Center (*m*/*z*)		Mass‐to‐charge ratio of peak maxima (*m*/*z*)
Center (migration time)		Migration time of peak maxima (min.)
		
Peak Height	*p* = max(*C*)	Maximum height of peak (counts)
Peak Volume	*∫C*	Total peak ion counts (counts)
Peak Width	*w* = ±3*σ*	Duration of peak (min.)
Z‐Score	$p/\max\left(\sigma_{B_{1}},\sigma_{B_{2}}\right)$	Signal‐to‐noise ratio
Gaussian loss	MSE(r, g)/*p*	Divergence from Gaussian profile (counts)
		
Background level	$\max\left(\hat{B_{1}},\hat{B_{2}}\right)$	Estimate of local baseline (counts)
Background SD	$\max\left(\sigma_{B_{1}},\sigma_{B_{2}}\right)$	Estimate of local noise level (counts)
Background diff	$\text{abs}\left(\hat{B_{1}}-\hat{B_{2}}\right)$	Delta between left and right baselines (counts)
Background ratio	$\min\left(\hat{B_{1}}/\hat{B_{2}},\hat{B_{2}}/\hat{B_{1}}\right)$	Ratio between left and right baselines

*Note*. $\hat{B_{1}}$ is the median of *B*_1_. $\sigma_{B_{1}}$ is the SD of *B*_1_. All calculations are performed after subtracting the local background.

#### 3.2.4 Peak Filtering

The specific peak properties and associated threshold values most informative for filtration of spurious peaks will strongly vary depending on CE‐MS instrument hardware, available mission downlink, the associated degree of filtration required, and to a lesser degree on specific target compounds of interest. Further, the filtration process should encourage science team trust through interpretability of its function and the parameters that control its behavior. To meet these operational requirements, ACME currently leverages an “expert system” comprised of simple threshold checks on the most informative peak properties. While this approach maximizes operator understanding and computational efficiency, there can be negative implications to mid‐mission reconfiguration and operator awareness of missed peaks as discussed in future work in Section 6.

ACME's peak filtration was optimized to the annotated training data set (Section 2.2.2) in a two‐step, semi‐automated manner. First, a decision tree model was trained to classify valid versus invalid peaks, and the model's reported feature importances were used to identify the most discriminative peak properties. For the OWLS CE‐MS prototype and Europa reference mission, the decision tree approach determined that *z*‐score, Gaussian loss, peak volume, and peak width were the most informative peak properties. Second, thresholds of acceptability for each property were manually determined (Table 3), incorporating both the reported values from the decision tree as well as domain knowledge from the instrument scientists. The resulting expert system rules are described in Algorithm 1.

**Table 3:** ACME Parameters for Algorithm Described in Section 3.2

Algorithm step	Parameter	Value
Background estimation	Window size	36 s
		
Gaussian convolution	Larger Gaussian SD	1.5 *m/z*, 2.9 s
	Smaller Gaussian SD	0.54 *m/z*, 1.0 s
		
Peak filtering	z‐score	>5
	Peak volume	>500 ion counts
	Peak width	>1.5 s @ z‐score > 10
		>2.4 s @ 10 > *z*‐score > 5
	Gaussian loss	<2%

Algorithm 1 **Determine if Peak is a Valid Target Peak**

1

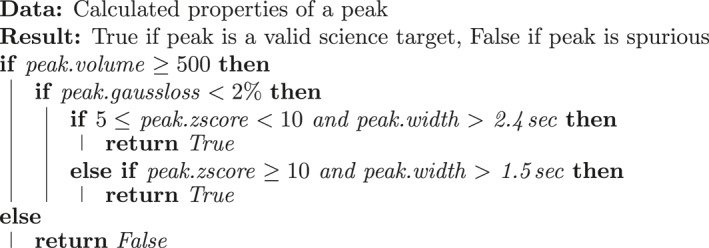

Should the data quality, compounds of scientific interest, or desired filtration rate change over the course of a mission, these rules can easily be reoptimized by repeating the above process with a new, representative, annotated data set. Should it be suspected that desirable peaks are being filtered, or that inappropriately high or low values of valid peaks are detected, mission operators may choose to downlink the entire pre‐filtration peak candidate list to inform reconfiguration of threshold values. Further, Section 6 describes future developments that will improve the robustness of ACME to flight failure scenarios featuring a probabilistic approach to peak validity.

### 3.3 Autonomous Science Data Products

ACME produces several autonomous science data products (ASDPs) that capture and summarize the contents of CE‐MS observations at a small fraction of the raw data volume to be compliant with the bandwidth restrictions of planetary exploration. Taken together, these products must first and foremost support the same valid science conclusions as would the full raw ion count grid. To do so, they must extract known key science observables (peaks), capture justifying context for interpretation of extracted quantities (background and data quality characteristics), support ground re‐analysis as new lines of inquiry arise (raw data crops), provide overlapping lines of evidence to support skeptical inquiry (overlapping information), reveal unanticipated structures in the raw data to support discovery (background), and provide insight into regions of non‐returned raw data to inform manual requests and identify the need for reconfiguration. Further, ACME must inform the later prioritization of multiple observations by estimating the scientific utility of a sample as well as identify key characteristics that reveal the uniqueness of a sample's contents. ACME can be reconfigured to include or exclude specific ASDPs or to adjust the size and resolution of each ASDP as required for a given mission use‐case. Detailed descriptions of ACME's output data products follow, and a product summary, as configured for the Europa reference mission, and is provided in Table 4. Note, that more expensive (higher data volume) products may be conditionally returned if the assessed priority of a sample's scientific content is high, again in a fully configurable manner.

**Table 4:** Overview of ACME's Autonomous Science Data Products (ASDP) That Would be Transmitted Depending on a Sample's Assessed Priority

ASDP	Data volume (kB)	Sample assessed as: low priority (kB)	Sample assessed as: high priority (kB)	
Valid peak properties^a^	3	✓		✓
Valid peak crops	108			✓
Compressed background	56			✓
Total ion count (TIC)	3	✓		✓
DD and SUE	0.3	✓		✓
Complete peak properties^a^, ^b^	(145)			
Raw data (for comparison)	(70,800)			
Total data volume per sample		6		170
Compression (raw/ASDP)		11, 800x		416x

*Note*. Data volumes are averages over the Testing Set.

^a^ Data product's size may be configured by selecting the peak properties to include.

^b^ Transmitted only upon special mission operator request.

#### 3.3.1 List of Detected Peak Properties

The primary science product from the CE‐MS data is a list of every peak candidate and its extracted properties as described in Section 3.2. During nominal operation, only valid peaks that survived filtration are downlinked to preserve bandwidth, and the complete list of peak candidates remains available onboard the spacecraft for later mission operator request should verification or reconfiguration of ACME's behavior be desired. In the event of a major discovery such as strong biosignature evidence, it is likely that the complete peak list would be requested to provide additional supporting context.

#### 3.3.2 Raw Peak Crops

**Figure 6:**
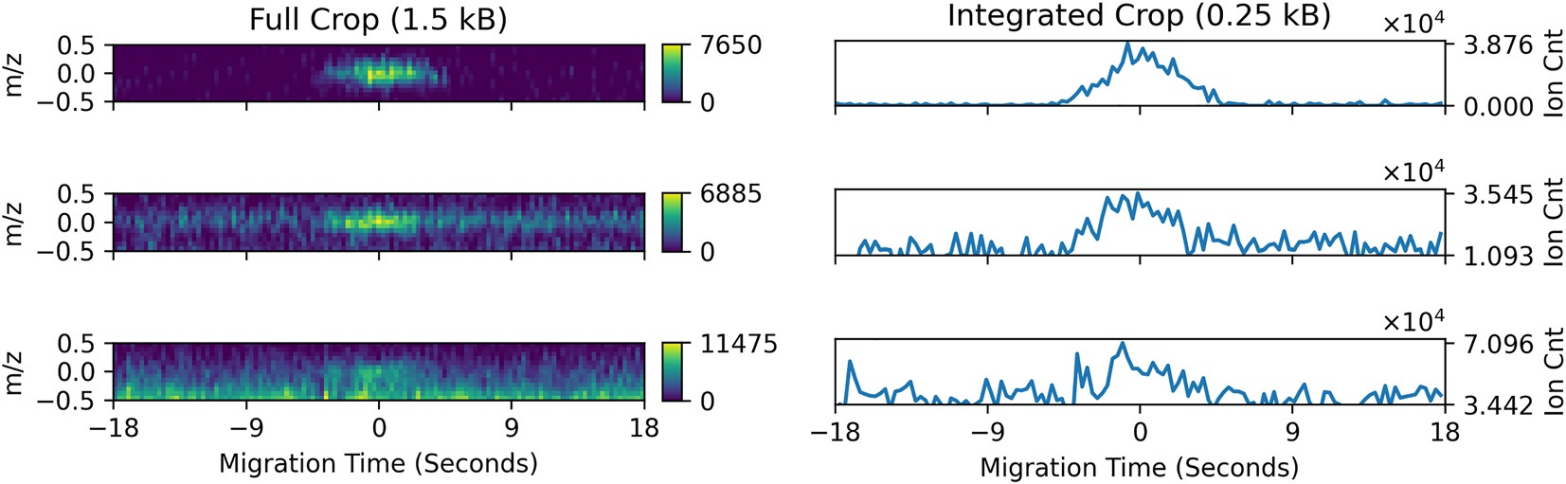
Full and *m/z*‐integrated crops for three peaks with decreasing SNR. Each 2D crop (left column) is centered on an identified peak and shows an area of 36 s by 1 *m/z* (121 time bins, 13 mass bins). The right column shows the same crops integrated over ±0.5 *m/z*. The ion count data was reduced to an eight‐bit representation to further preserve bandwidth. Returning raw data regions allows independent assessment of ACME's findings as well as enables later re‐tuning of ACME's settings.

For each valid peak, ACME captures a window from the surrounding raw ion count data. Selected raw data regions are crucial to enable ground science analysis and interpretation. This is the most expensive data product by volume, and its size is configured by specifying the desired window size and the level of peak candidate filtration. Additional data volume savings may also be realized by optionally reducing the bit depth of the returned ion counts and/or integrating over the *m/z* dimension to instead return local electropherograms. Bandwidth protection is ensured by a specified maximum number of peaks to receive raw data capture. Figure 6 shows three example raw peak cropped regions as well as their integrated alternatives.

#### 3.3.3 Background Characterization

**Figure 7:**
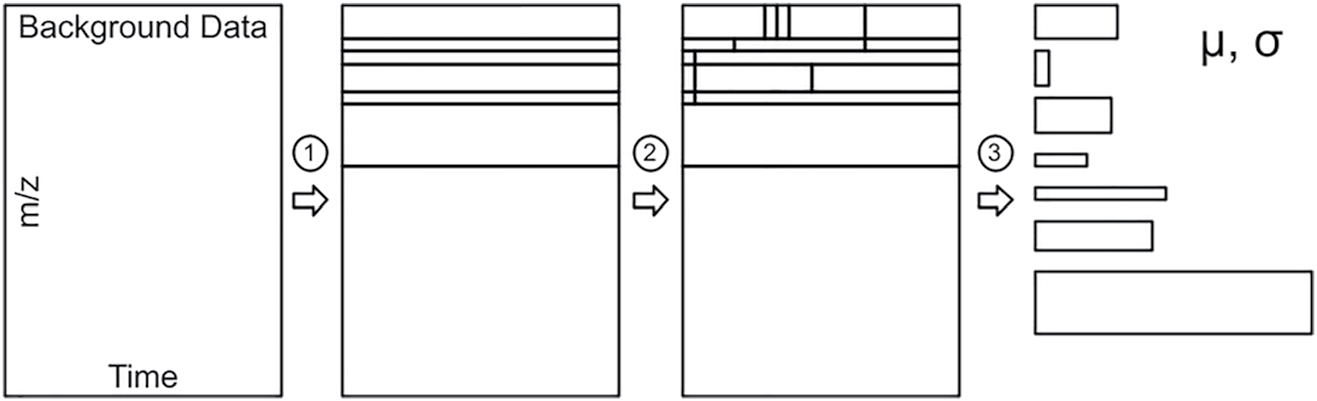
Diagram of the ion count background summarization method. (1) Identify horizontal edges in the background noise for segmentation, (2) In each segment, identify vertical edges for further segmentation, (3) For each region, calculate and store the mean and SD.

While the list of peaks properties, associated properties, and cropped raw data regions well characterize the primary science observables of a CE‐MS instrument, ACME must ensure that unanticipated or serendipitous discovery remains possible even if not peak‐like in nature (e.g., broad, flat peaks, see Section 4.4). Further, characterizing the complex background enables detailed instrument health monitoring, provides context for peak detection decisions, and supports reasoning on potentially undetected peaks. To efficiently parameterize the CE‐MS background using a minimum amount of data bandwidth, ACME leverages the observation that the ion count background can be efficiently described by a series of rectangular regions with fairly uniform noise behavior. These subregions typically extend broadly (several minutes) in time and narrowly (0.5–2 *m/z*) in *m/z*. To enable background characterization, ACME dynamically determines subregion boundaries and captures its mean and standard deviation. The region determination algorithm is diagrammed in Figure 7.

The background summarization product may be configured by changing the segmentation percentile thresholds, resulting in coarser or finer representation of the background structure. Alternatively, to enable a more uniform data product size, ACME can produce multiple background summarization products at differing threshold values and select the highest resolution product that satisfies a maximum allowed data volume.

#### 3.3.4 Total Ion Count

ACME separately reports the total ion content integrated over the full *m/z* and migration time ranges, corresponding to a mean mass spectra and electropherogram. This product provides context for ACME's more detailed findings, insight into instrument health, as well as ensuring that large but highly non‐Gaussian peaks are still captured. Configuration options include down‐sampling the series and reducing its bit depth to preserve bandwidth. Down‐sampling by a factor of 4 and an eight‐bit encoding results in an average data volume of 3 kB for the testing set.

### 3.4 Prioritization‐Supporting Output Products

While onboard summarization enables high data volume instruments to be deployed on remote worlds, potential discoveries that are environmentally rare may still be missed by the relatively limited number of summarized samples that may be returned. Detailed characterization of an unknown environment requires representative sampling that returns the most diverse set of observations as well as the distribution, or rarity, of each example. Prioritization is the capability to determine which subset of available samples would be most scientifically informative to return. ACME generates two products that enable sample‐level prioritization. The science utility estimate (SUE) prioritizes observations based on a mission's explicit science goals, while the diversity descriptor (DD) focuses on rare or unusual observations and enables inter‐sample similarity comparisons. These two synergistic approaches may be later combined into a single prioritization scheme in any ratio, allowing a science team to configure and reconfigure ACME's prioritization behavior to best match the science team's current goals.

#### 3.4.1 Science Utility Estimation for Known Science Targets

The SUE is a real‐valued estimate (ranging from 0 to 1.0) of the scientific value of a CE‐MS observation as defined by the known science targets of the mission use‐case. The SUE is constructed from the extracted information produced by the summarization algorithms in Section 3.3. The precise formula is highly mission use‐case specific and may evolve over the mission lifetime as new findings might motivate to focus on other signals. To support this need, ACME includes a variety of potentially useful inputs to consider for inclusion in a given SUE instantiation. Following data science nomenclature, we call these extracted inputs “features” of the observation. Each of the candidate features captures a separate, desirable property of a valid, scientifically interesting CE‐MS observation, such as the number of peaks in a specified mass and time region of interest or an estimate of the instrument data noise level. The set of features of interest for SUE calculation will generally be determined during mission preparation and will likely not change until later phases of the mission or in the event of unanticipated data quality issues.

Each raw input feature, *x*_*i*_, is normalized and conditioned for SUE calculation by the transformation:

(1) $$y_{i}=\left\{\begin{array}{cc} \sqrt{\frac{x_{i}}{x_{i,\max\;}}} & x_{i}<x_{i,\max\;}\\ 1 & x_{i}\geq x_{i,\max\;} \end{array}\right.$$

where *x*_*i*,max_ is a user defined threshold for each input feature that sets its maximum significant contribution. The square root dependence below this threshold encourages rapid initial contributions that gradually taper toward saturation and diminishing returns.

The SUE is calculated by a weighted average over the set of N normalized features:

(2) $$\mathrm{SUE}=\frac{\sum_{i=1}^{N}w_{i}*y_{i}}{\sum_{i=1}^{N}w_{i}}$$

where *w*_*i*_ are user‐specified weights corresponding to the relative importance of each feature.

For the prototype OWLS CE‐MS and the Europa reference mission, five features were chosen for SUE calculation to demonstrate observation prioritization. (a) Priority compound presence, as defined by the number of valid peaks with *m/z* values matching a customizable onboard list of high‐priority organic compounds (e.g., amino acids, nucleobases, nucleosides), (b) Observation complexity, as defined by the total number of identified target peaks, (c) Observation SNR, as defined by the average *z*‐score of identified target peaks to prioritize high compound abundance, (d) Number of unique compounds as estimated by the number of unique migration times to 36 s accuracy, and (e) Number of unique compounds as estimated by the number of unique *m/z*'s to 1 AMU accuracy. Table 5 summarizes these features and their associated saturation thresholds and weighted importance for SUE calculation. Future missions may easily subsample from this list, use any other extracted features of interest, or adjust the weights and saturation values.

**Table 5:** Demonstrated Features for the Science Utility Estimate

Feature	Saturation (*x*_*imax*_)	Weight (*w*_*i*_)
Number of target organic compound peaks	100	1
Total identified peaks	200	0.5
Average *z*‐score (peak height/background noise)	100	0.2
Number of unique migration times	50	1
Number of unique *m*/*z*	100	1

#### 3.4.2 Diversity Descriptors for Representative Sampling

The needs of planetary scientists to characterize an unknown environment extend beyond the stated science targets of interest. Besides the need for confirmatory repeat observations, diversity‐based sampling requires to calculate the dissimilarity of a CE‐MS observation relative to those already onboard or returned to Earth. Observations that strongly differ from those previously seen may receive an increased priority relative to those that are highly similar to past observations. Further, even for the observations that are not prioritized for downlink and remain onboard, their unique contributions may be summarized to the ground such that their similarity to the returned observations is known.

In ACME, both prioritization by observation uniqueness as well as comparison with nondownlink‐selected observations are enabled by the choice of a scalar distance metric that measures the dissimilarity between two CE‐MS observations. The features that enter this distance metric are called the observation's diversity descriptor (DD) and form a vector whose elements capture observation details that are meaningfully comparable. Similar to the SUE, the raw input features, *x*_*i*_, are normalized and thresholded similar to the SUE:

(3) $$y_{i}=\left\{\begin{array}{cc} \frac{x_{i}}{x_{\mathrm{imax}}} & x_{i}<x_{\mathrm{imax}}\\ 1 & x_{i}\geq x_{\mathrm{imax}} \end{array}\right.$$

The relative difference, *D*, between two observations, *a* and *b*, with *N* DD elements can then be calculated by the normalized Euclidean distance between their respective DD vectors:

(4) $$D\left(a,b\right)=\sqrt{\frac{\sum_{i=1}^{N}{\left(\left(a_{i}-b_{i}\right)*w_{i}\right)}^{2}}{\sum_{i=1}^{N}w_{i}^{2}}}$$

where the user‐defined weights, *w*_*i*_, define the relative importance of each DD feature.

For the prototype OWLS CE‐MS and the Europa reference mission, three features were selected to enable diversity‐based sampling through the DD. The first two estimate salt abundance as (a) the average background ion count and (b) the SD of the background in the proximity of peaks. To enable compound‐level diversity sampling, the final feature (c) is a vector itself and captures the binary presence of peaks (1 or 0) for coarsely binned *m/z* (10 AMU resolution). The features and weights used are provided in Table 6.

**Table 6:** Features Used to Calculate the Diversity Descriptor

Feature	Saturation (*x*_*imax*_)	Weight (*w*_*i*_)
Average background height	50	0.2
Standard deviation of background height	50	0.2
Peak presence in *m/z* bins^a^	1.0	1.0^b^

^a^ Feature is a vector.

^b^ Cumulative weight of vector elements.

In summary, the scalar SUE and the vector DD are calculated for each observation, using a variety of mission‐specific extracted features and user‐defined weights. Together, they enable later data prioritization by estimating the science utility relative to known mission science targets and the uniqueness of each sample's contents.

**Figure 8:**
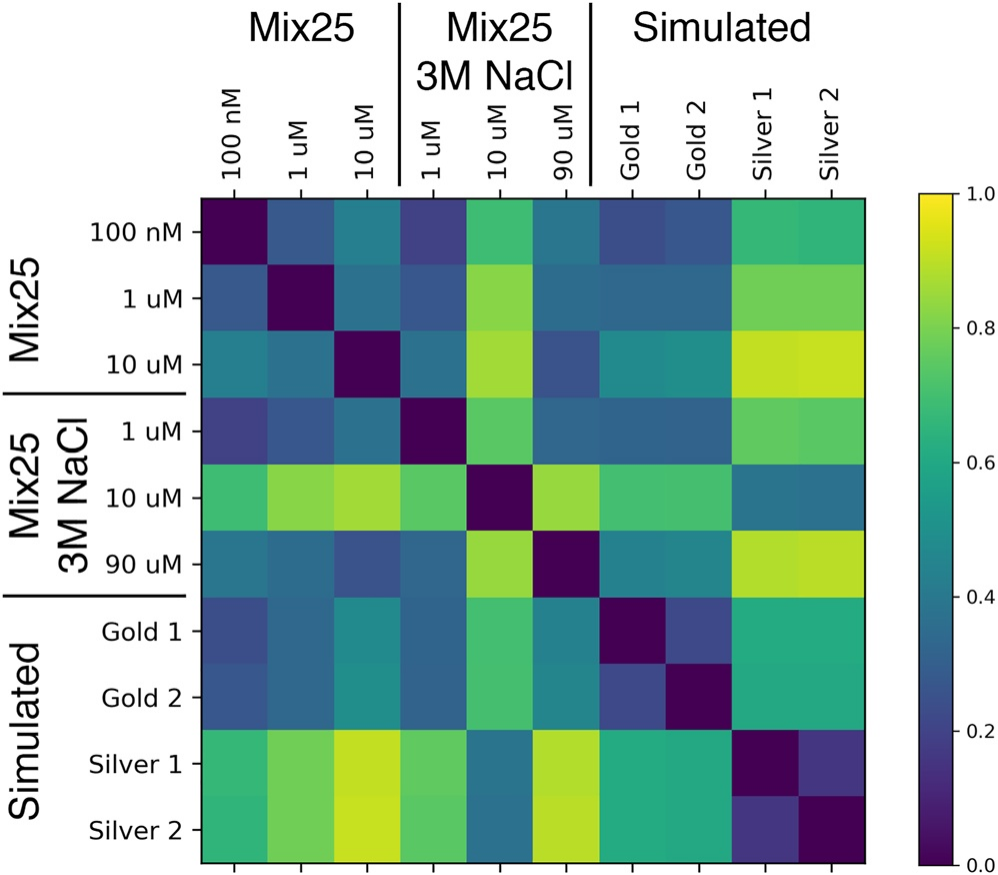
Dissimilarity, that is, Euclidean distance, D, between six laboratory samples and two samples from the Golden and Silver simulated data set (see Section 2.2.3) as determined by their diversity descriptors (DD's) and user defined weights, *w*_*i*_ (Equation 4). Zero corresponds to two samples being exactly the same, 1 corresponds to two experiments being the most different.

As an illustrative example of assessing observation dissimilarity, the data sets of Section 2.2 were compared in Figure 8. The laboratory and simulated data sets are visibly grouped together by similarity, with the exception of 10 μM Mix25 NaCl 3M. The simulated “Golden” and “Silver” data sets are similar within their respective data set but differ significantly to each other.

#### 3.4.3 Downlink Prioritization

Downlink prioritization for the OWLS instrument suite is accomplished by balancing two competing goals: (a) selecting data products with high science utility for downlink, and (b) selecting a diverse set of representative data products that capture the full range of observed phenomena. This is achieved in a two‐step process. First, data products are ordered by the highest SUE‐per‐byte value, in order to achieve the maximal utility within a budgeted downlink data volume. Second, the DD is used to penalize observations by their similarity to observations higher in the priority list, to account for the decreased marginal utility of downlinking similar data products (Doran et al., 2021) except for repeat confirmations. The magnitude of the DD penalty, and hence the relative weight of target science (SUE) versus representative sampling (DD), is user‐defined and easily modified by mission operators.

In addition to ACME's downlink prioritization, scientists maintain the ability to manually request and prioritize observations for downlink during sequencing and ground‐in‐the‐loop commanding opportunities. For example, this allows to prioritize repeat confirmation observations in case an earlier sample indicated signs of life. Manual control over prioritization is enabled by preserving the common mission practice of “priority bins.” In this scheme, operators specify which data products to place in each of several priority bins. Products from higher‐priority bins are always downlinked ahead of lower‐priority bins until the communication opportunity ends, ensuring interpretable, predictable downlink behavior. ACME's SUE‐ and DD‐based prioritization algorithm would only apply to observations within each bin. This hybrid prioritization strategy emphasizes informing the operations team and leveraging their guidance when available, while ensuring a reasoned, predictable, and productive behavior otherwise. In the extreme case of an extraordinary discovery captured by ACME's prioritized, summarized data products, it is likely that a follow‐on, manual request for transmission of the raw observation data would be made. ACME, its summary data products, and its prioritization strategies have been designed to ensure that the mission science operations team is sufficiently informed to make such a request.

## 4 Results and Discussion

### 4.1 Target Peak Detection

Three data sets were used to evaluate the peak detection performance of ACME. The “Golden” and “Silver” simulated data sets (see Section 2.2.3) were used to characterize ACME's performance under controlled and ideal conditions, while laboratory test samples (see Section 2.2.1) were used to evaluate ACME under more realistic conditions for ocean worlds.

ACME's peak detection performance was captured by the metrics of precision and recall. Precision is the fraction of peaks ACME selected that were valid, annotated target peaks, that is, how likely is an ACME‐selected peak to be valid. Recall measures the fraction of valid, annotated target peaks in the data set that were correctly selected by ACME, that is, how many of the known targets were correctly detected. Both of these metrics are critical to the space use‐case. Low precision would result in false peak detections that would corrupt SUE and DD prioritization, and the unnecessarily captured raw data regions surrounding uninteresting peaks could crowd out science targets from the downlink record if present in large numbers. More directly, however, a low recall would result in missed scientifically relevant peaks, erroneously diminished SUE prioritization, and seriously compromise the goal of identifying signals of existing life. Due to the high scientific cost of missing target peaks, ACME was optimized to emphasize recall over precision. During the initial decision tree modeling described in Section 3.2.4, the model received a higher penalty for false negatives than for false positives. On the Golden and Silver data set, ACME has a recall and precision of greater than 0.99 and 0.98, respectively. For the six hand annotated samples of the test set, the average precision and recall is 66% and 99%, respectively. The recall and precision for the simulated and laboratory samples is summarized in Table 7.

**Table 7:** Performance of ACME for Simulated and Laboratory Samples

Data set	Precision	Recall
Golden simulated data set	0.99	0.99
Silver simulated data set	0.98	0.98
Laboratory test samples^a^	0.66	0.99

^a^ Average of six samples in “testing data set” (Table 1).

#### 4.1.1 False Positives

**Figure 9:**
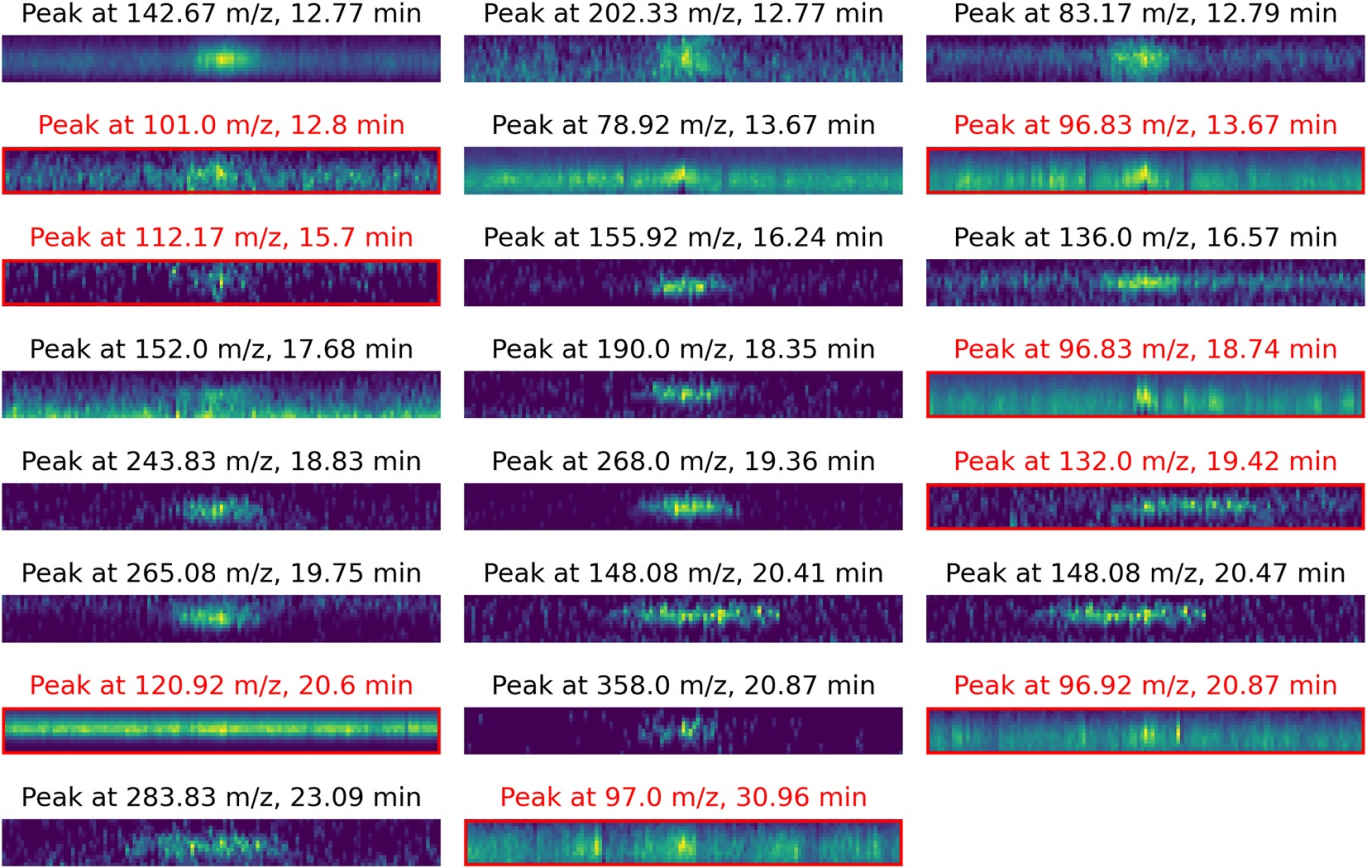
Cropped regions of peaks detected in the 100 nM Mix25, no salt test sample with the ACME software. Each peak shows an area of 36 s by 1 *m/z*. False positive peaks are highlighted in red.

**Figure 10:**
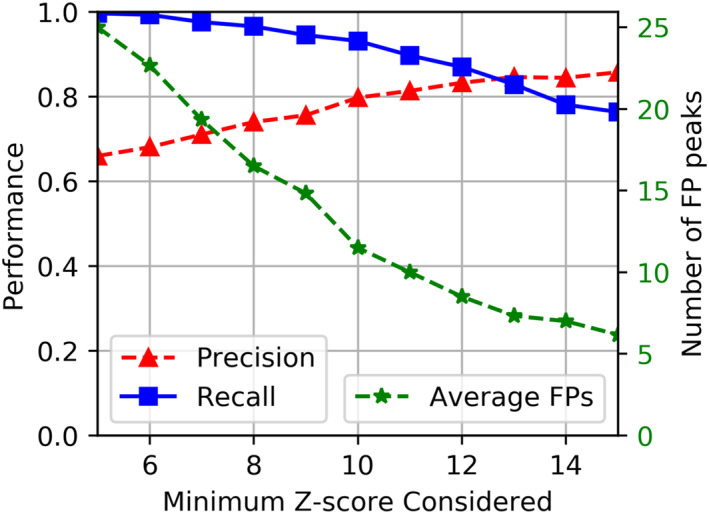
Filter study for ACME's z‐score threshold on hand labeled laboratory samples. ACME's performance is shown for different z‐score thresholds (*x*‐axis) used to detect peaks. The recall is shown with blue squares, precision with red triangles (*y*‐axis on the left), and average false positive (FP) rate per sample with green stars (*y*‐axis on the right).

We consider any peaks detected by ACME that were not labeled as such by instrument scientists to be false positives. A moderate number of false positive peaks (moderate precision) have little negative impact, as they would result in unnecessary raw cropped region capture and potential inflation of the observation priority. However, substantial false positives (low precision) may crowd out valid science targets of interest. As mission bandwidth decreases, the sensitivity to false positives increases. Figure 9 captures cropped regions surrounding some of ACME's false positive peaks extracted from our evaluation data, highlighted with a red border. Many of these peaks share similar characteristics to true positive peaks (e.g., the false positive peak at 101.0 *m/z* and 12.8 min; left column, second from the top). Should peak‐like structures be present as unwanted artifacts in the data, optimizing ACME to reject them will also remove weaker true positive peaks and thus reduce overall sensitivity. This tuning trade‐off is captured in Figure 10. As sensitivity is lowered by increasing the required *z*‐score threshold for peak detection, the number of false positives decreases (green) but so too does the recall of valid peaks decrease (blue). A specific mission use‐case will enable further optimization in the tuning of ACME.

### 4.2 Background Summarization

ACME's background summarization algorithm (Section 3.3.3) was evaluated by comparison with two baseline image compression methods.

#### 4.2.1 Baseline Methods

Two common, off‐the‐shelf image compression algorithms used for science imagery were selected as a comparison baseline. JPEG2000 (Rabbani, 2002) is a lossy compression method based on wavelet decomposition typically used for planetary mission camera images (Kiely & Klimesh, 2003). The principal component analysis (PCA) dimensionality reduction method (Pearson, 1901) instead focuses on preserving data covariance through the creation of a reduced basis set. For both of these methods, the CE‐MS 2D ion count grid was first scaled and discretized to an eight‐bit integer image (0–255). The image was then compressed and reconstructed for comparison with ACME, with configuration parameters set to produce results similar in size to ACME's ASDPs.

#### 4.2.2 Reconstruction Comparison

**Figure 11:**
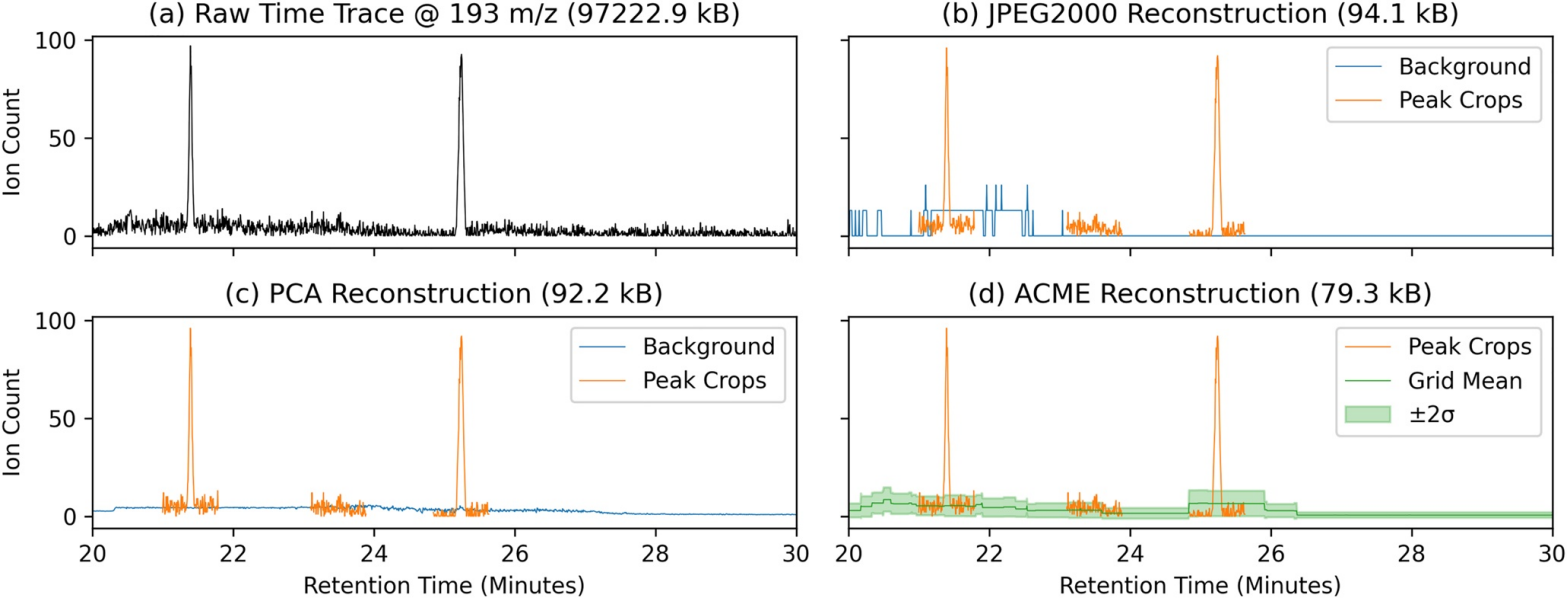
A comparison of reconstructions from three different methods (JPEG2000, PCA, ACME). For comparison with raw data, crops of detected peaks have been overlaid on the reconstructed background in orange. The data sizes reported are of the entire CE‐MS observation.

ACME's background summary product was compared to the baseline compression methods. An example is shown in Figure 11 for the 90 μM Mix25 NaCl 3 M laboratory sample. All three methods reduced the 97 MB CE‐MS raw observation by roughly three orders of magnitude, but with greatly differing scientific fidelities. To provide comparison and context, three cropped regions of raw data were overlaid in orange. The JPEG2000 background reconstruction in Figure 11b shows clear artifacts due to the scaled integer discretization that is a necessary preprocessing step by the method. The PCA reconstruction in Figure 11c better captures the average ion count background around the peaks, but it fails to capture the local noise estimate (SD). Characterization of the noise is critical for analyzing the validity of a peak detection (SNR). ACME's summarization product shown in Figure 11d solves this issue by summarizing both the mean and variance of the background. With these two pieces of information, it is possible to conclude that the detected peak at 23.5 min is most likely a false positive, as the height of the “peak” is within the summarized noise range.

### 4.3 Computational Efficiency

Constrained onboard computation bandwidth constraints will be ubiquitous features of a mission to Europa or Enceladus. To maximize communication opportunities and mission productivity, the ACME system is required to process a new observation within a 30 min time frame. On a standard laptop CPU (Intel i9 2.4 GHz) the ACME algorithm required 70 s (average over the six test‐set samples), operating on a single core, to process one raw CE‐MS observation and produce all specified ASDPs. The OWLS instrument suite has currently targeted a processor that meets or exceeds the Qualcomm Snapdragon 801 that was successfully flown on the Ingenuity helicopter (Balaram et al., 2021) on the Perseverance rover. This processor can reach 2.5 GHz for each of its four cores (Qualcomm, 2021). Thus, we expect the onboard processing time to be similar to our tests on the standard laptop and exceed the mission computation requirement. As a reference for comparison, the processing time on a radiation hardened CPU, RAD750 V2 at 200 MHz (BAE Systems, 2017), would be approximately 14 min and still meet the requirement.

### 4.4 Limitations

#### 4.4.1 False Negatives

Separating true peaks from the noisy background requires making a variety of assumptions. ACME currently assumes that peaks can be approximated by a Gaussian distribution. This holds true for the vast majority of analyzed laboratory peaks. However, in the OWLS CE‐MS development data, there exist two examples of non‐Gaussian peaks.

**Figure 12:**
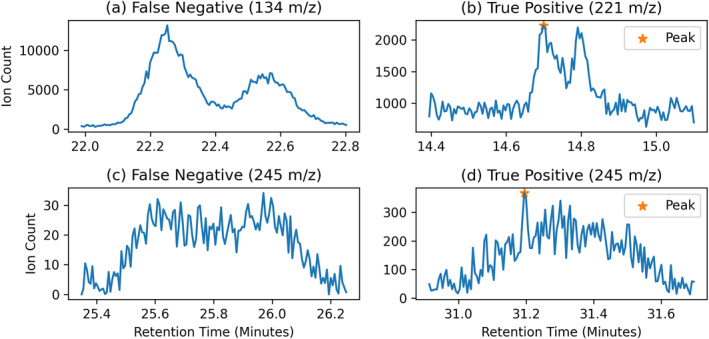
Examples of four false negative scenarios. (a) Two overlapping peaks not detected by ACME because they cannot be estimated by a single Gaussian distribution. (b) Two closely overlapping peaks that can be approximated by a single Gaussian distribution. (c) A very wide peak that was not detected by ACME due to its similarity to background artifacts. (d) A slightly less wide peak which was correctly detected by the algorithm.

**Figure 13:**
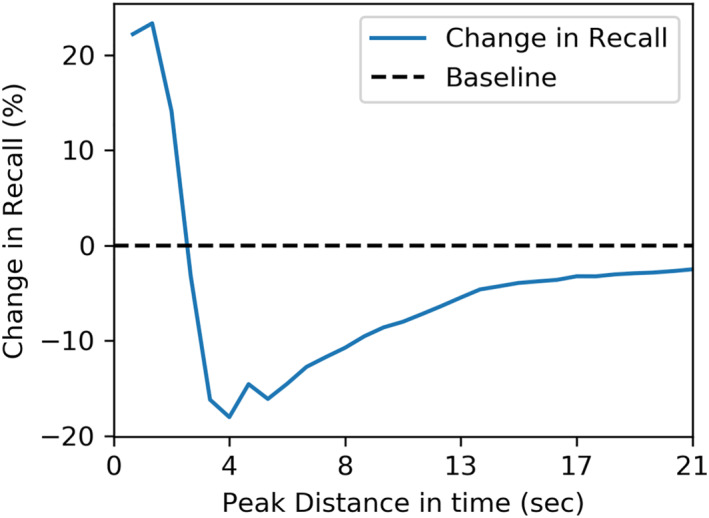
ACME peak detection as two peaks converge and overlap. Baseline recall is shown as the black dotted line, while the change in recall is shown in blue. As the peaks overlap, recall suffers, until they become sufficiently close that they increase the chance of including each other in their cropped raw data regions.

The first type occurs when two peaks are in close proximity in the time domain and overlap partially. The ACME software correctly detects some of these cases as two peaks; however, in other cases only one of the peaks (Figure 12b) or no peak (Figure 12a) is detected. We quantified the effect of peak overlap on algorithm recall using a set of simulated data containing a total of 10,000 peaks with a peak‐width range of (1–27 s) (see Figure 13). As shown, the recall for a target peak decreases due to overlap to a minimum of about −20% at a distance of 4 s. Any closer, and recall increases compared to the baseline to approximately +20%. This can be explained by the algorithm only being required to correctly detect one of the two peaks. The other peak is then close enough to be counted as detected, even if a filter removed it, for example, due to its z‐score or Gaussian fit. While this second peak would be missed in the list of peaks, it would be contained in the raw data crops.

The second type of non‐Gaussian peaks are very long with a plateau on top that can span more than 30 s, as shown in Figure 12c. These peaks approximate other background artifacts and are not detected in some instances. Although ACME can be retuned to be more sensitive to these cases, a resultant rise in false positives would be anticipated. These very long peaks are very rare in the CE‐MS observations and considered out of scope for the ACME use case at this time.

#### 4.4.2 Failure Modes

It is crucial that even if ACME's configuration is misaligned with respect to the nature of incoming CE‐MS observations, mission operators will be able to recognize and reconfigure ACME to restore functionality. This section captures ACME's modes of failure and ensures that operators would have access to both timely alerts and decision supporting information on how to proceed.

There are a variety of low‐sensitivity scenarios in which target peaks would be partially or completely missed (see Section 3.1). Should ACME erroneously detect few or no target peaks despite their presence, the SUE would be near 0, and only the valid peak properties list, TIC, DD, and SUE products would be transmitted due to low prioritization. However, the TIC would still capture an integration of the ion counts that revealed the presence of undetected peaks, alerting the operations team of an issue with ACME configuration. Should multiple samples contain no target peaks, the operations team would be able to manually request the list of complete peak properties and background summary, retune ACME, and resolve the situation with the uplink of the new configuration at the next communications opportunity. The peak list and background would provide ample information to assess the cause for the misalignment, whether it be instrument related or unanticipated environmental character.

Similarly in the over‐sensitive limit, ACME could erroneously detect unacceptably large numbers of peaks, presumably either spurious background peaks in an unexpectedly large noise environment or through real unexpected environmental complexity. This would be problematic, as raw data crops surrounding each peak would exceed the downlink bandwidth allocated to ACME. To mitigate this scenario, ACME has an adjustable threshold for the maximum number of identified target peaks per observation. If ACME's detected peaks exceed this threshold, the observation is treated as low priority due to data quality issues, and again only the TIC, DD, and SUE will be transmitted for further analysis along with an operator alert. The mitigation of this scenario proceeds as in the low sensitivity scenario above.

Finally, the ACME background summarization method (Section 3.3.3) leverages that the background ion count patterns are aligned to the *m/z* and migration time axes in the data. For highly complex, unaligned background noise, the resulting data product would exceed its maximum downlink allocation of 100 kB. In such a scenario, ACME may be configured to fall back on the JPEG2000 image compression algorithm, as this approach makes no morphological assumptions and supports specifying the maximum size of the resulting compressed image. This would allow operators to view the overall CE‐MS observation structure even in the event that it radically departs from expectation, likely due to instrument issues.

## 5 Conclusion

To search for life on distant ocean worlds, a flight‐like CE‐MS instrument with associated onboard capabilities for scientific summarization and prioritization has been developed. The large data volumes of the mass spectrometer, coupled with the limited downlink budget of a distant planetary mission such as Europa or Enceladus, will require science autonomy software. These onboard autonomy capabilities must maximize ground operator awareness of the raw data onboard and provide justification for its decisions. The ACME software can autonomously identify peaks in noisy CE‐MS data, compress the analyzed experiments by up to three orders of magnitude, and quantify the uniqueness and estimated science utility of each observation. These configurable capabilities enable the return of data products that maximize target science capture as well as characterization of a diverse, unknown environment, even in the presence of severe downlink constraints. ACME has been shown to meet or exceed the expected performance requirements for the Europa Lander reference mission (Tan‐Wang & Sell, 2019).

## 6 Future Work

Several improvements to ACME are currently in progress as part of the continuing OWLS project. The current ACME product was designed to support initial instrument hardware development, terrestrial field demonstrations, and later extend to the space mission use‐case. These improvements further support and refine planetary science applications.

### 6.1 Compound‐Level Assessment

Currently, the SUE of an observation relies partially on the number of unique peak *m/z* and migration time values. These are used as approximate estimates for the number of unique molecular compounds present in an observation. Instead, compounds could first be identified along the time axis and then associated with their mass fragmentation spectra. This would improve the quality of compound number estimation, and would also support prioritization by comparison with an onboard library of known, high‐priority compounds of interest.

### 6.2 Peak Detection Likelihood

Currently, ACME filters spurious from valid peaks using a binary filter with simple threshold checks. This approach was selected for maximum operator understanding and trust of the science team during instrument development and initial field deployment. However, a peak candidate that has low SNR may indeed be a detection of interest, rather than a spurious noise event. A more nuanced approach would assess each peak candidate by its likelihood of validity, such as with a Gaussian process classifier. Then, given a fixed budget of *N* raw cropped regions informed by bandwidth constraints, the first *N* peak candidates sorted by likelihood would be captured in detail. Additional, peaks with sufficiently high likelihood could still be returned along with their extracted properties, but without the expensive cropped region. This would more naturally mitigate the failure modes identified in Section 4.4.2 by affording mission operators sufficient information to dynamically adjust ACME's parameters without the costly request of the entire peak candidate list in the next uplink opportunity.

### 6.3 Uncertainty Quantification

ACME must engender trust from mission science teams and flight operators to provide meaningful, useful insights into the environmental exploration science target, the instrument's health and data quality, and its own function and calibration. As in the peak likelihood example above, adding an estimate of uncertainty to ACME's core products can afford deeper operator insight and more correctly capture the contents of observations for summarization and prioritization. Both the SUE value and the DD elements could, for example, include uncertainties. This would allow several new lines of onboard reasoning. For example, a high priority observation with significant uncertainty may be less preferential than a slightly lower priority observation with low uncertainty. Observations that result in highly uncertain SUE and DD elements could be effectively flagged as violating ACME's fundamental assumptions and instigate operator inquiry. ACME's current background summarization method already incorporates this concept by capturing the variance in each background ion count region. Current R&D efforts will explore the mission utility and interpretability provided by adding uncertainty quantification to the various ACME output products.

### 6.4 Internal Calibration and Diagnostics

Future versions of the OWLS CE‐MS instrument will include an internal standard consisting of known compounds at known concentrations. These standard compounds may be processed by the CE‐MS and ACME before natural samples. ACME's performance on these internal standards could then be compared to an expected outcome to provide a host of instrument and autonomy health information critical to interpreting the natural sample results. ACME's ability to efficiently summarize a CE‐MS observation will be critical for these standards, to ensure a minimum of downlink bandwidth is spent on calibration information. In the event of instrument degradation for missions with very short lifespans or limited communication opportunities, such as the Europa Lander reference mission (Tan‐Wang & Sell, 2019), it may be desirable to further equip ACME with a form of auto‐tuning that would determine its optimal peak sensitivity based on the results from the internal standard. Additionally, will simulate the effects of radiation noise to characterize and compensate any effects on the science objective. This research task would be especially important for a high radiation environment like Europa. We will simulate bit flips in the data that represent instrument detector hits or in‐memory modifications of observations following (Granat et al., 2009). The precise effects on extracted characterizations will depend on how these bit flips materialize. Some will produce easily recognizable “spikes” that can be neglected, while others will more subtly distort peak characteristics.

### 6.5 Natural Sample Characterization

As ACME represents a data‐driven approach to onboard science support, it is only as trustworthy as the observations that have been used to develop and validate its capabilities. Currently, the majority of ACME's evaluation was performed on samples prepared in a laboratory environment with a fixed list of compounds relevant to the search for extant life. Upcoming terrestrial field campaigns with the OWLS instrument suite will provide uncontrolled natural samples to be assessed by ACME and, in turn, assess ACME's summarization and prioritization capabilities. The final evaluation of ACME's relevance will be obtained by comparing the scientific findings produced by two groups of scientists. One group will have access to the raw CE‐MS observations, while the other will only see ACME's bandwidth‐compliant ASDPs and prioritization results. This will mock‐up an actual mission use‐case and inform further improvements in ACME's operational design.

## Data Availability

The open source ACME software is implemented in Python 3 and available at: https://github.com/JPLMLIA/OWLS-Autonomy (Machine Learning and Instrument Autonomy Group, Jet Propulsion Laboratory, 2022). The data used to develop and validate ACME are available at: https://doi.org/10.5281/zenodo.5849873 (Mauceri et al., 2022).
